# Differential detection of *Ascaridia galli* and *Heterakis gallinarum* eggs in intestinal and caecal excreta of floor-housed laying hens: a longitudinal study

**DOI:** 10.1017/S0031182025101534

**Published:** 2026-02

**Authors:** Teka Feyera, Brendan Sharpe, Isabelle Ruhnke, Stephen W. Walkden-Brown

**Affiliations:** 1 Animal Science, School of Environmental and Rural Science, University of New England, Armidale, NSW, Australia; 2 New South Wales Department of Primary Industries and Regional Development, Armidale, NSW, Australia; 3 Livestock Clinics – Division of Poultry, Faculty of Veterinary Medicine, Freie Universität Berlin, Berlin, Germany

**Keywords:** chickens, diagnosis, egg count, nematodes, poultry

## Abstract

*Ascaridia galli* and *Heterakis gallinarum*, the most prevalent nematodes of chickens, inhabit the small intestine and caeca, respectively, and often co-occur. Current excreta egg count (EEC) methods do not differentiate between their eggs, and although chickens produce two distinct excreta types – intestinal excreta (IE) and caecal excreta (CE) – the distribution of eggs of these species across them remains poorly understood. Forty Hy-Line Brown laying hens (40 weeks, mean body weight (BW) 2·07 ± 0·02 kg), cleared of prior nematode infection and artificially infected with *A. galli* (n = 20) or *H. gallinarum* (n = 20) were housed in separate floor pens and monitored for 26 weeks. Assessments included clinical signs, EECs from IE, CE and mixed excreta (ME), and worm recovery from subsets of birds at 8, 14, 20 and 26 weeks. Neither infection resulted in clinical signs, but *A. galli* slightly reduced BW gain (0·5 g/week/hen) than *H. gallinarum* (2·8 g/week/hen). Egg detection aligned with worm predilection sites: *A. galli* eggs were predominantly found in IE, while *H. gallinarum* eggs were largely confined to CE. In ME samples, egg counts were reduced by 45% relative to IE for *A. galli* and 60% relative to CE for *H. gallinarum*. EECs showed a negative but non-significant association with excreta moisture content. Natural re-infection produced a stable adult worm population in both infections. These findings demonstrate that analysing IE and CE separately provides a practical, non-lethal approach for differentiating these infections, while ME appears to have limited diagnostic utility. Further studies should evaluate these patterns across broader conditions and individual variation.

## Graphical abstract

**Figure:**
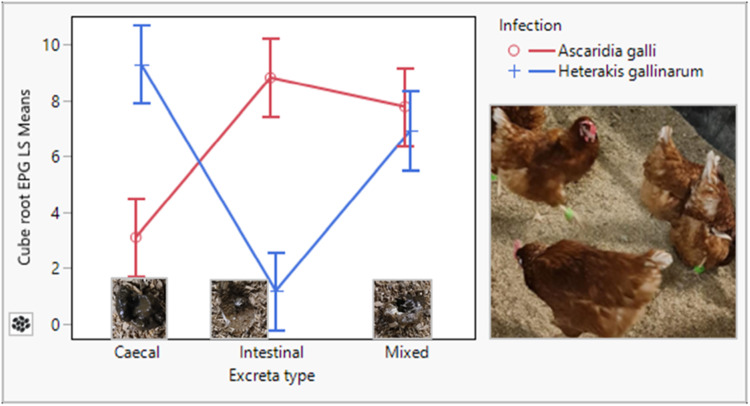

## Introduction

The ascarid nematodes *Ascaridia galli* and *Heterakis gallinarum* are the most common and economically important gastrointestinal nematodes in laying hens (Shifaw et al., 2021b). These nematodes differ in their adverse effects on chickens, with *A. galli* being usually more pathogenic (Permin and Hansen, 1998; Gauly et al., 2001). They have different predilection sites in the gastrointestinal tract of chickens, where *A. galli* resides in the small intestine whilst *H. gallinarum* invades the caeca (Permin and Hansen, 1998). They are characterized by a lifecycle involving excreta deposition of eggs into the environment via the excreta, followed by larval development in the egg under suitable conditions until the infective stage is reached. This is followed by ingestion of the egg by the host (or paratenic hosts in the case of *H. gallinarum*) with further development of larvae within the host and growth to adulthood and commencement of egg production (Soulsby, 1982; Permin and Hansen, 1998). *A. galli* has a longer prepatent period (5–8 weeks) than *H. gallinarum* (4–5 weeks) (Ramadan and Abouznada, 1992). Co-occurrence of these nematodes is very common in cage-free commercial poultry operations (Shifaw et al., 2021a). Differentiating the level of infection with these pathogens provides challenges in the field, currently requiring bird sacrifice or necropsy of culled chickens to assess worm burdens in the two predilection sites. Because the eggs of these two species are very similar (Permin and Hansen, 1998; Tarbiat et al., 2021) routine worm egg counts in excreta currently do not provide information on the relative burdens of the two species.

Worm egg count determination is a cornerstone of nematode infection monitoring in the livestock industries, even in this era of genomics, metagenomics, proteomics and bioinformatics (Demeler et al., 2012; Nielsen, 2021). This is true for the poultry industry, where excreta egg count (EEC) is a useful tool to indirectly estimate adult worm burdens of a host animal as positive relationships between worm burden and EEC have been demonstrated in mono-infections, particularly during the patent period (Permin and Ranvig, 2001; Daş et al., 2011b; Feyera et al., 2021; Shifaw et al., 2022).

Interpretation of EEC data for diagnostic purposes in mixed infections is complicated by the fact that the eggs of *A. galli* and *H. gallinarum* cannot be easily differentiated morphologically (Permin and Hansen, 1998; Zloch et al., 2021). While there are some morphological differences between the eggs of these nematodes, differentiation using copromicroscopic methods requires professional skills and trained personnel (Tarbiat et al., 2021). Specifically, *A. galli* eggs are slightly larger and more oval with a smoother shell surface, while *H. gallinarum* eggs are slightly smaller, rounder and possess a thicker, more textured shell (Höglund et al., 2023). However, the two species inhabit different parts of the gastrointestinal tract and chickens and other birds have complex gut physiology that results in separation of intestinal excreta (IE) and caecal excreta (CE) into easily identifiable droppings (Duke, 1989; Rychlik, 2020). *H. gallinarum* resides in the caeca, which are two blind-ended sacs that project from the proximal colon at its junction with the small intestine (Clench and Mathias, 1995). The CE is the main transport-medium for eggs of *H. gallinarum* to the external environment (Daş et al., 2019) and could potentially serve a diagnostic role for detecting *H. gallinarum* infection. While an earlier study (Fine, 1975) reported that *H. gallinarum* eggs are shed almost exclusively in CE, the proportional distribution of each species’ eggs across different excreta types has not been formally quantified in controlled single-species infections. A recent study in Australia reported that EECs were similar in the two types of excreta collected from 16 laying flocks harbouring mixed ascarid infections (Shifaw et al., 2023). It is common for chicken farmers to monitor nematode infections using fresh excreta samples collected from the floor of the housing facility, which do not represent samples from individually identified birds. Laboratory analysis results are usually reported simply as ‘ascarid eggs’, especially in mixed infections (Oladosu et al., 2023; Shifaw et al., 2023).

Apart from the technical limitations, several other factors may influence the reliability of egg counts in relation to worm burden. For instance, excreta consistency can skew EEC results; for example, diarrhoea increases excreta moisture, which dilutes the number of worm eggs (Le Jambre et al., 2007). Furthermore, parasites egg excretion quantity may vary from hour to hour (within the day) or from day to day (between days) due to endogenous or exogenous factors. In most cases, nematode eggs are shed at a higher rate during the day, particularly late afternoon or early morning (Villanúa et al., 2006; Wongrak et al., 2015; Daş et al., 2019). Egg excretion by chicken nematodes *A. galli* and *H. gallinarum*, for example, has been shown to follow a diurnal fluctuation pattern (Wongrak et al., 2015; Daş et al., 2019), with significant variations within and between egg excretion days (Villanúa et al., 2006). Therefore, all parasites of a host do not simultaneously contribute to the countable eggs in a single sample of excreta droppings. This applies particularly to parasites that reside in physically separated organs such as the caeca (Villanúa et al., 2006). Therefore, these factors must also be considered not only when determining EEC but also when sampling animals for this purpose.

It has been proposed that burdens of *A. galli* and *H. gallinarum* could be estimated from egg counts in IE and CE, respectively (Fine, 1975; Sherwin et al., 2013; Feyera et al., 2022). IE is passed 12–15 times a day (approx. every 2 hrs) and is normally firm, consisting of digested matter and partially covered by white crystals of urate. CE is passed infrequently (approximately twice a day). It comprises the evacuated contents of the caecum, and is soft, smelly and light brown, can be foamy and soft creamy with no urates or undigested matter (Clench and Mathias, 1995; Cupo and Beckstead, 2019). It is highly likely that the worm eggs in each of the two types of excreta predominantly originate from the two different nematode species (Fine, 1975; Cupo and Beckstead, 2019). However, cross-contamination cannot be excluded.

This study aimed to determine the degree of contamination of IE and CE by ascarid eggs in chickens having mono-specific infections of *A. galli* and *H. gallinarum* and kept under a floor husbandry system. This will indicate whether this can serve as a practical tool for the differential diagnosis of these two species, whose co-occurrence is very common in the field (Shifaw et al., 2021b). The study also assessed how the type of excreta influences the measured eggs per gram (EPG).

We hypothesized that specific infections with *A. galli* and *H. gallinarum* result in egg detection markedly skewed towards presence in the IE and CE, respectively, and that this will be sufficient under field conditions to differentiate between infections with the two species. Under this general hypothesis, we tested the following specific propositions: i) In chickens infected with *A. galli* alone, worm eggs would be detected almost exclusively in the IE rather than the distinct CE; ii) In chickens infected with *H. gallinarum* alone, the distribution of worm eggs will be heavily skewed towards their presence in the CE; iii) In ascarid mono-infections, determining worm egg counts in random mixed intestinal-caecal excreta (ME) will underestimate the measured EPG level relative to counts in pure excreta types.

## Materials and methods

### Experimental birds and management

Forty Hy-Line Brown laying hens, 35 weeks old, were sourced from a commercial farm in Tamworth, NSW, Australia. At induction, the birds were treated with a double dose of short-acting anthelmintics (Levamisole at 56 mg/kg and Fenbendazole at 10 mg/kg) to eliminate any existing nematode infection. Treatments were delivered into the crop via gavage needles at a dose rate calculated based on body weight of the individual birds. Absence of infection was confirmed by testing of IE and CE every second day between 10 and 20 days post-treatment while the birds were kept in individual cages. The hens were then stratified by body weight and allocated to two experimental groups; each was placed in a separate floor pen. Birds were then orally infected with 450 embryonated eggs of either *A. galli* or *H. gallinarum*, administered in three split doses over one week. The study protocol was approved by the animal ethics committee of the University of New England (UNE) (approval number ARA24-049).

### Infectious materials

Infectious eggs were obtained from mature female *A. galli* and *H. gallinarum* worms isolated from naturally infected free-range chickens. Fresh small intestine and caeca were separated and opened to collect the worms by rinsing the contents through sieves (500 mm for *A. galli*, 250 mm for *H. gallinarum*). *A. galli* is a large, thick-bodied nematode found in the small intestine, with females reaching up to 110 mm and males around 76 mm. *H. gallinarum* is smaller, inhabits the caeca, with females measuring about 13–15 mm and males 7–10 mm. The collected worms were then sexed by morphological characteristics under dissecting microscope, and sexually mature females were isolated and rinsed several times with tap water and used for worm egg recovery (Stehr et al., 2018).

For *A. galli*, preparation of the infectious material (egg isolation and embryonation procedures) was as described previously (Feyera et al., 2020). Briefly, freshly harvested *A. galli* worms were transferred into RPMI media (with 0·1% 100 units/mL penicillin, 100 μg/ml of streptomycin, 250 ng/mL amphotericin B; Sigma-Aldrich Pty Ltd, St Louis, USA) in a glass jar to a volume that covered the worms. The *A. galli* worms were then maintained for 3 days at 37°C, changing the media every 24 h. After every 24 h, the media containing parasite eggs were collected into 50 mL screw cap falcon tubes, and the jar was rinsed with fresh RPMI media. The egg suspension was then centrifuged (Beckman Coulter Inc, Brea, USA) at 425 *g* for 1 min, and eggs concentrated at the bottom of the media were collected using transfer pipettes. Eggs were subsequently resuspended in 0·1 N H_2_SO_4_ and incubated aerobically at 25°C for 4 weeks. For *H. gallinarum*, preparation of the infectious material (egg isolation and embryonation procedures) was done as described previously (Gauly et al., 2008; Püllen et al., 2008). Mature female worms were dissected with scissors and then squeezed gently by rubbing a pestle on a plastic tea strainer in a mortar to release the eggs into a petri dish. The body tissues were first coarsely removed through sieving with the tea strainer, and the eggs were then rinsed with tap water through a series of sieves with mesh aperture sizes of 100 and 75 mm. Finally, the eggs were collected in a 36-mm sieve as previously described (Gauly et al., 2008). Freshly collected eggs were then incubated in 0·1% potassium dichromate (K_2_Cr_2_O_7_) for approximately 4 weeks at 25°C (Gauly et al., 2008; Püllen et al., 2008).

In both cases, a small quantity of the incubation medium was added occasionally to maintain a constant volume, and the egg cultures were aerated continuously with container lids open. Eggs of each species were maintained in their specific media and never exposed to low temperatures (i.e. not stored in a refrigerator). On the day of infection, the eggs of *A. galli* and *H. gallinarum* were separately rinsed in a 36-mm sieve and collected in a saline solution (NaCl, 0·9%) at room temperature. The eggs were assessed several times to determine the percentage of embryonated eggs by morphological classification, as described by Feyera et al. (2020).

### Experimental outline and sampling approach

#### Experimental infection

Prior to inducing infection, full embryonation of the infective ascarid eggs was assessed and confirmed microscopically using a compound binocular microscope equipped with a digital Nikon H550S camera (Nikon Corporation, Tokyo, Japan). Only eggs demonstrating a coiled larval stage were classed and counted as viable/embryonated (Rahimian et al., 2016; Feyera et al., 2020). For inoculation, embryonated eggs were diluted with tap water to generate the desired concentration (embryonated eggs/mL) containing the infection dose to be given to each bird. The number of eggs/mL suspension was determined with a modified McMaster method using the Whitlock universal egg counting chamber (Whitlock, 1948). All the birds in the respective infection group were separately inoculated with 450 embryonated eggs of *A. galli* or *H. gallinarum* in 3 split doses over a week. The infected birds were then reared in group floor pens, with wood shavings as bedding material, until the end of the experiment.

#### Excreta collection and analysis

**Figure 1.**
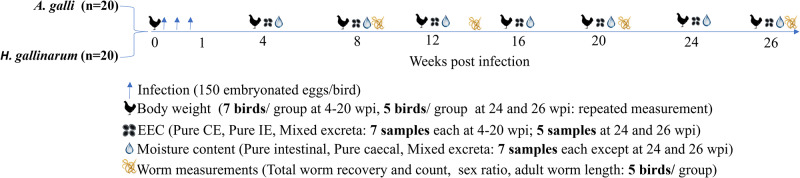
Experimental outline and timeline of main experimental events and measurements for evaluating the level of detection of *A. galli* and *H. gallinarum* eggs in intestinal and caecal excreta of barn-housed laying hens. EEC = excreta egg count expressed as eggs per gram of excreta (EPG); wpi = weeks post infection; IE = intestinal excreta; CI = caecal excreta. Some of the pictorial elements in this figure were generated using an AI tool (**Illustrate AI)**.

**Figure 2.**
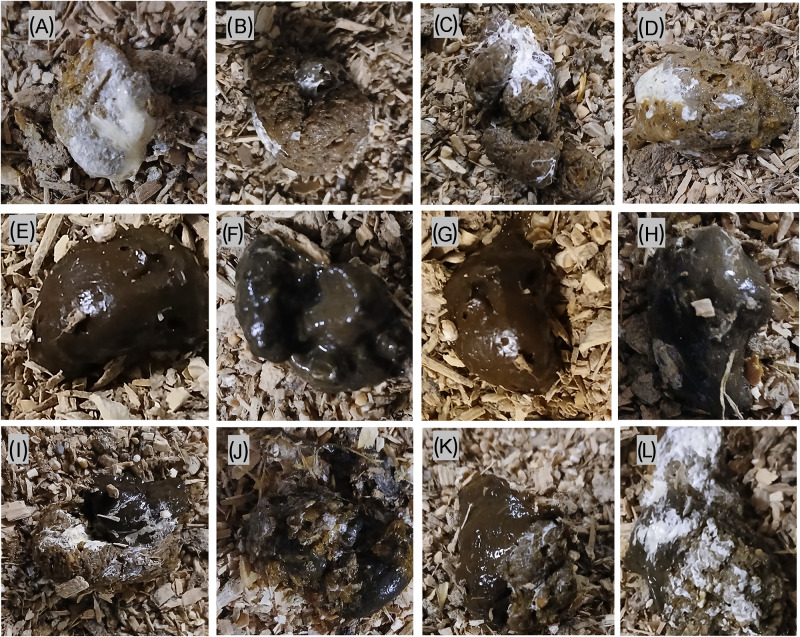
Representative examples of pure intestinal (A–D), pure caecal (E–G), and mixed intestinal-caecal (I–L) droppings collected from the floor of barn-housed laying hens harbouring *A. galli* and *H. gallinaraum* specific infections.

**Figure 3.**
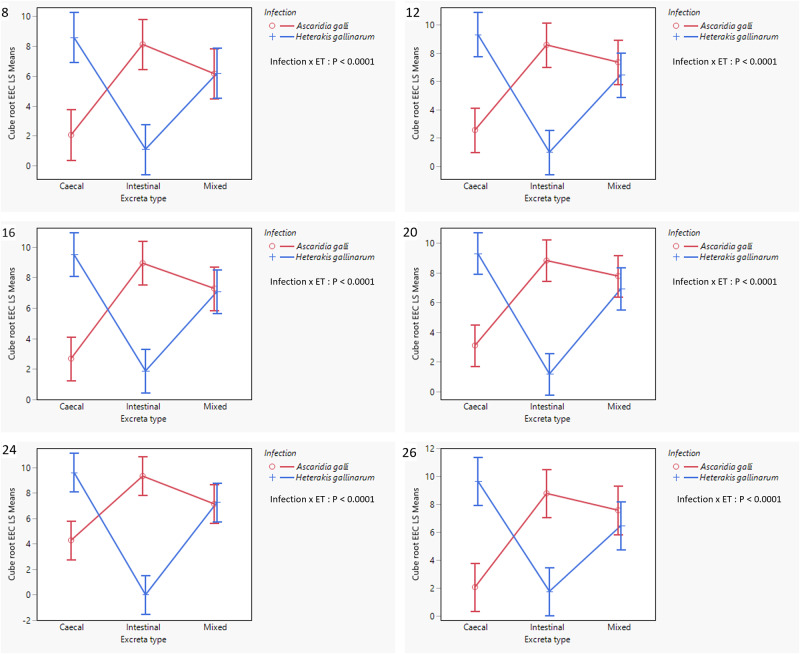
Interaction plots (LSM ± SE) of cube root transformed EECs showing interaction between infection and excreta type in *A. galli* and *H. gallinarum* mono-infected hens. The plots in order represent data obtained at 8-, 12-, 16-, 20-, 24- and 26-weeks post-exposure to infection. ET = excreta type, LSM = least square means, SE = Standard error, EEC = excreta egg count.

The experimental outline and timeline of main experimental events and measurements are depicted in Figure 1. The study aimed to mimic practical aspects of on-farm regular nematode infection monitoring by egg farmers in a flock of laying hens. The birds in each infection group (n = 20) were kept on floor pens for 26 weeks without changing their litter material. Animal rearing and management were in such a way that it allowed for continuous exposure to faecal-oral cycling of infections to mimic the field floor-based housing husbandry system. The birds were monitored for clinical signs of disease every day and sampled at regular intervals to assess the level of infection and degree of detection of parasite eggs in excreta samples. As primary subjects of this study, three types of excreta samples were defined, collected, and analysed for worm egg counts and moisture content at 4-week intervals. These were: 1) grossly pure IE with no visible mixing (contamination) with CE (Figure 2A–D), 2) grossly pure CE with no visible mixing (contamination) with IE (Figure 2E–H) and 3) Excreta samples in which both intestinal and caecal droppings were clearly mixed (ME) during defecation (Figure 2I–L).

For this purpose, 7 (individual) samples of each excreta category (a total of 21 fresh samples) were collected from the floor of each infection group at each sampling time point, except at 24 and 26 weeks post-infection (wpi), where only 5 samples of each category were collected per group due to the reduced number of birds at this stage. For worm eggs detection and enumeration, the modified McMaster techniques with a limit of detection of 40 EPG was employed (Whitlock, 1948). In brief, 2·5 g of each excreta sample was diluted in 47·5 ml of saturated salt solution (S.G. = 1·20), thoroughly homogenized, sieved and a 0·5 ml aliquot loaded into a chamber on a Whitlock universal slide (JA Whitlock and CO, Eastwood, NSW, Australia) and examined under 40x magnification power. Eggs counted were multiplied by 40 to provide an EEC in EPG units.

The excreta samples studied did not represent samples obtained from individually identified animals. Excreta collection was conducted from early morning to early evening to ensure that each dropping came from individual birds as much as possible, minimizing the potential of obtaining more than one sample from a single animal at a specific sampling time point. In situations where sufficient samples of the CE and ME categories were not collected, sampling was conducted the following day in the same manner. Thus, it is highly likely that IE, followed by CE, were sourced from different individual animals (at a specific sampling time point), whereas there is a possibility that mixed-type samples may have been sourced from the same animal, especially when two days of sampling were involved.

In addition to worm egg counts, each sample was also subject to dry matter analysis to determine the moisture content. Briefly, 2–5 g of excreta samples of each category placed in crucibles were weighed. The samples were then placed in 105°C oven overnight (24 hrs), and their dry weight was recorded. The moisture content of the samples, expressed as a percentage, was then calculated as 100-(dry sample weight (g)/wet sample weight (g)*100).

#### Clinical observations and body weight

The chickens were inspected daily at the same time for clinical signs of infection (loss of appetite, drooping wings, ruffled feathers, diarrhoea or mortality) following the criteria of Permin and Hansen (1998). Individual body weights were recorded using an electronic scale on day 0 and then every 4 weeks, aligned with coprological analysis, until the end of the experiment at 26 weeks post-infection (wpi) (Figure 1). Seven individually tagged birds were weighed repeatedly at each time point, except at 20 and 26 wpi, when only 5 birds were recorded.

#### Necropsy and worm recovery

Five hens were humanely killed from each group at 8, 14, 20 and 26 wpi for the determination of total worm load, worm population composition, worm sex ratio and worm size. Worm recovery and burdens were determined according to standard laboratory methods consistent with guidelines of the World Association for the Advancement of Veterinary Parasitology (WAAVP) (Yazwinski et al., 2022). For the examination, the intestines including both caecal tubes were opened longitudinally using Mayo scissors. Contents of each section were sieved with a 100 μm mesh sieve, flushed with tap water and all visible worms retained on the sieve were collected and counted then the remaining sample and the scraped mucosa in a Petri dish containing saline were examined under a stereomicroscope (40x) to count immature and microscopic worms. Adult *A. galli* and *H. gallinarum* worms were then sexed and body lengths determined. Worm sex was determined using helminthological keys: Female *A. galli* worms are longer than males having a straight posterior terminal, whereas males have a curved posterior terminal. Male *H. gallinarum* worms have a straight tail with dissimilar spicules, whereas females have a long, narrow and pointed tail (Permin and Hansen, 1998; Yazwinski and Tucker, 2008; McDougald, 2020).

### Data analysis

Data were statistically analysed using JMP^®^ software version 18·0 (SAS Institute Inc., Cary, NC, USA). Distributions of the data and model residuals were assessed for compliance with the assumptions of analysis of variance (ANOVA). Body weight of chickens, worm length and moisture content of the excreta did not require transformation. EECs were cube root transformed whereas worm counts (female, male and larvae numbers, sex ratio and total worm burden) were log transformed prior to data analysis to better meet the assumptions. Temporal changes in body weight were analysed using repeated measures analysis with individual bird fitted as a random factor in a REML (restricted maximum likelihood) model and infection type, wpi and their interaction fitted as fixed effects. EEC and moisture content of the excreta samples were analysed within each sampling time fitting effects of infecting species (infection), excreta type and their interaction. Worm recovery data (counts and sex ratio) at each sampling time were analysed by 1-way ANOVA fitting the fixed effect of infecting species (infection). Changes in worm load over time for each species were analysed separately by fitting the fixed effect of wpi. Worm lengths, expressed in cm/worm for *A. galli* and mm/worm for *H. gallinarum* were analysed separately for each species within each sampling time. Tukey’s HSD was employed to test for significant differences between levels of a given factor in the analysis. Artificial infection establishment rate (worm recovery per bird at 8 wpi/infection dose x100), and proportion of excreta samples positive for worm eggs were presented using descriptive statistics. Linear regression analysis was used to test the association between different parasitological variables and the rate of change thereof. Statistical significance was set at *P* < 0·05 for all analyses.

## Results

### Confirmation of treatment application, clinical findings and body weight change

The success of anthelmintic treatment to clear prior infections was confirmed by repeated negative EEC in all birds prior to artificial infection. Monospecific infections were successfully induced in each chicken as detailed in the sections below and in no bird was the non-infecting species detected. No mortality was recorded during the experiment and except for sporadic mild diarrhea in a few chickens, the birds exhibited no visible clinical signs of helminthiasis throughout the observation period. At the start of the trial, the mean body weights of hens in the *A. galli* and *H. gallinarum* infection groups were 2·01 ± 0·05 and 1·99 ± 0·06 kg, respectively, and at the completion they were 2·05 ± 0·06 and 2·08 ± 0·07 kg, respectively.

Despite continuous exposure, neither infection type (*P* = 0·35) nor time post-infection (*P* = 0·76) had a significant effect on body weight changes over time. However, hens harbouring *A. galli* infection persistently experienced a slightly lower body weight gain compared to *H. gallinarum* infected hens over the course of the infection period, with growth rates of 0·52 (y = 2044·7 + 0·52x) and 2·18 (y = 2055·6 + 2·18x) grams per week, respectively.

### Differential detection of worm eggs in different types of excreta

At 4 wpi, only 2/42 excreta samples contained worm eggs, both being CE samples from *H. gallinarum* infected hens. Thereafter, all IE from *A. galli* infections and all CE from *H. gallinarum* infections were consistently positive for worm eggs, with ME positive in 97·4 and 100% of cases, respectively (Table 2). Table 1 presents the effects of infection and excreta type on the level of EEC at each sampling wpi, and Figure 3 illustrates the significant interaction between the effects of infection and excreta type. For both experimental infections, EEC increased until 12 wpi, after which it stabilized or exhibited minor fluctuation.

**Table 1.** Analysis of worm egg counts (LSM ± SE) in hens harbouring ascarid mono-infections by type of infection and type of excreta sample at different weeks post exposure to infection

	Weeks post infection													
	4		8		12		16		20		24		26	
Factor	EEC ± SE	AM	EEC ± SE	AM	EEC ± SE	AM	EEC ± SE	AM	EEC ± SE	AM	EEC ± SE	AM	EEC ± SE	AM
Overall Mean ± SE	0·18 ± 0·12	2·85	5·37 ± 0·34	334	5·87 ± 0·32	408	6·23 ± 0·23	433	6·19 ± 0·28	425	6·26 ± 0·29	456	6·05 ± 0·34	417
Effect and Level														
**Infection**	*P* = 0·13		0·82		0·37		0·80		0·18		0·04		0·80	
*A. galli*	0·00 ± 0·17^a^	0·00	5·45 ± 0·48	350	6·15 ± 0·45	426	6·30 ± 0·41	428	6·57 ± 0·39	449	6·90 ± 0·42^a^	461	6·14 ± 0·.48	416
*H. gallinarum*	0·37 ± 0·17^a^	5·71	5·30 ± 0·48	318	5·58 ± 0·45	390	6·16 ± 0·41	438	5·8 ± 0·39	401	5·62 ± 0·42^b^	450	5·96 ± 0·48	418
**Excreta type**	*P* = 0·12		0·18		0·03		0·05		0·0067		0·0034		0·12	
Intestinal	0·00 ± 0·21^a^	0·00	4·62 ± 0·59	322	4·77 ± 0·55^b^	485	5·41 ± 0·49^b^	422	6·20 ± 0·49^ab^	385	4·65 ± 0·52^b^	412	5·86 ± 0·59^b^	388
Caecal	0·55 ± 0·21^a^	8·57	5·33 ± 0·59	374	5·93 ± 0·55^ab^	474	6·10 ± 0·49^ab^	485	5·00 ± 0·49^b^	468	6·94 ± 0·52^a^	536	5·27 ± 0·59^ab^	488
Mixed	0·00 ± 0·21^a^	0·00	6·18 ± 0·59	305	6·90 ± 0·55^a^	365	7·19 ± 0·49^a^	391	7·35 ± 0·49^a^	422	7·19 ± 0·52^a^	420	7·02 ± 0·59^a^	376
**Infection x Excreta type^a^**	*P* = 0·12		*P* < 0·0001		*P* < 0·0001		*P* < 0·0001		*P* < 0·0001		*P* < 0·0001		*P* < 0·0001	

EEC ± SE represent cube root transformed and statistically analysed data. Where superscripts are shown values in the same column within each experimental factor and level not sharing common superscripts (a, b) are statistically different. EEC = excreta egg count, AM = arithmetic mean, SE = standard error.

a Interactions are shown in Figure 3.

There was little difference in overall EEC between the two infections, with significant differences observed only at 24 wpi (*P* = 0·04). In contrast, excreta type had a more pronounced influence on EEC with significant differences observed at 12, 16, 20 and 24 wpi due mostly to higher values for mixed excreta within the factorial combination.

More importantly, as hypothesized, there was significant interaction between the effects of infection and excreta type at all sampling wpi during the patent infection period (8–26 wpi, *P* < 0·0001; Figure 3). As clearly shown in Figure 3, *A. galli* infection resulted in significantly higher EEC in IE, followed by ME, with CE having the lowest counts. In contrast, an opposite trend was observed in *H. gallinarum*-infected hens, with CE samples having the highest EECs and IE the lowest. In ME samples, EECs were intermediate between the two excreta types, but tended to be higher than the mean of the other two excreta types, as also shown in Table 3. Relative to the highest EECs observed for *A. galli* and *H. gallinarum* in IE and EE, respectively, there tended to be a greater reduction in EEC in the mixed samples (a dilution effect) from *H. gallinarum* infections.

Table 2 presents additional descriptive worm egg detection data from the three excreta sample types obtained from 8 to 26 wpi. The data show that *A. galli* eggs tended to contaminate CE more than *H. gallinarum* eggs contaminated the IE, both in terms of proportion of positive samples and EEC level. Out of 38 IE and CE samples examined for each nematode species, 52·6% of CE harboured *A. galli* eggs, whereas only 23·7% of IE samples harboured *H. gallinarum* eggs in mono-specific infections. In hens harbouring *A. galli*, the mean EEC values were 747 and 92, respectively, in the IE and CE (8-fold higher in IE). For *H. gallinarum*, the corresponding values were 23 and 842, respectively (36-fold higher in CE). EEC values in ME were 45% lower than values in IE for *A. galli* infected hens and 60% lower than values for CE for *H. gallinarum* infected hens.

**Table 2.** Descriptive summary of the differential detection of *Ascaridia galli* and *Heterakis gallinarum* eggs in intestinal, caecal and mixed excreta samples collected from hens with ascarid mono-infections between 8 and 26 weeks post-infection, showing the proportion of egg-positive samples and descriptive statistics of excreta egg counts

			Descriptive statistics of EEC (eggs per gram of excreta)		
Infection	Excreta type	Proportions of samples positive for worm eggs^a^	Mean	Median	Range
*A. galli*	Caecal	52·6% (20/38)	91·6	40	0–440
	Intestinal	100% (38/38)	747·4	700	120–1760
	Mixed	97·4% (37/38)	422·1	400	0–760
*H. gallinarum*	Caecal	100% (38/38)	842·1	760	360–1600
	Intestinal	23·7% (9/38)	23·2	0	0–160
	Mixed	100% (38/38)	334·7	320	80–760

a Samples collected at 4 wpi (n = 7 per each excreta type per infection) were not included as only 2 CE samples from *H. gallinarum* infection were positive for worm eggs, and this period does not represent a fully patent infection. EEC = excreta egg count.

### Moisture content of the droppings and association with worm egg count

The mean moisture content was 77·6 ± 0·29% (71·8–86) for IE, 80·3 ± 0·29% (74–85·2) for CE, and 78·9 ± 0·29% (71·6–84·9) for ME. Moisture content did not differ significantly between infection types at any sampling time. No significant association was found between moisture content and EEC, although weak, non-significant negative associations were observed for IE (*P* = 0·13) and CE (*P* = 0·30), but not ME.

### Worm establishment, burden and composition over time

Table 3 presents worm population composition, total worm load and adult worms sex ratio over time. The worm recovery data showed that all birds harboured mono-specific infections. Despite being given the same infective dose, a considerably higher infection establishment rate (number of adult worms at 8 wpi per 450 eggs × 100) was observed for *H. gallinarum* (29·1%) compared with *A. galli* (2·49%). At all sampling weeks, *H. gallinarum* infection resulted in significantly higher counts (*P* < 0·001) of both adult worms and larvae than *A. galli* infection. Total worm count tended to generally increase with time (*P* < 0·05) for measurements during the patent period (> 8 weeks) in both infection types but with no significant difference after 8 wpi. In both cases, the change in worm load was mainly attributed to the significant increase in larvae count (*P* < 0·05) as the number of adult worms showed little change (*P* > 0·05). Adult worms predominated throughout all sampling times, accounting for 72–95% in *A. galli* and 71–93% in *H. gallinarum*, with female-to-male ratios remaining close to parity (0·97–1·32 and 0·99–1·07, respectively).

**Table 3.** Total worm recovery (count/bird) and population composition over time in hens experiencing continuous re-infection to *A. galli* and *H. gallinarum* worms expressed as arithmetic mean ± SE

		Weeks post infection			
Worm count	Species	8	14	20	26
Male	*A. galli*	5·20 ± 5·12^b^	8·80 ± 7·54^b^	9·80 ± 10·2^b^	7·80 ± 6·97^b^
	*H. gallinarum*	65·2 ± 5·12^a^	71·2 ± 7·54^a^	73·2 ± 10·2^a^	72·6 ± 6·97^a^
Female	*A. galli*	6·00 ± 5·10^b^	10·4 ± 5·67^b^	9·60 ± 10·7^b^	7·40 ± 5·99^b^
	*H. gallinarum*	65·8 ± 5·10^a^	69·8 ± 5·67^a^	77·4 ± 10·7^a^	71·2 ± 5·99^a^
Larvae	*A. galli*	0·60 ± 3·20^b^	7·20 ± 6·54^b^	9·60 ± 10·4^b^	8·0 ± 6·41^b^
	*H. gallinarum*	9·60 ± 3·20^a^	55·4 ± 6·54^a^	57·4 ± 10·4^a^	45·2 ± 6·41^a^
TWC	*A. galli*	11·8 ± 10·9^b^	26·4 ± 15·9^b^	29·0 ± 28·4^b^	23·2 ± 17·0^b^
	*H. gallinarum*	140·6 ± 10·9^a^	196·4 ± 15·9^a^	208·0 ± 28·4^a^	189·0 ± 17·0^a^
Sex ratio	*A. galli*	1·33 ± 0·22	1·22 ± 0·14	1·07 ± 0·09	0·97 ± 0·15
	*H. gallinarum*	1·01 ± 0·22	1·01 ± 0·14	1·08 ± 0·09	0·99 ± 0·15

Values are arithmetic means (± SE) but statistical analysis was performed using transformed data. Values in the same column, within each observation week, not sharing common superscripts (a, b) are statistically different. wpi = weeks post infection; TWC = total worm count; SE = standard error.

Data for male and female worm lengths are given in Table 4. The analysis showed that worm sizes followed the established trend of larger females and smaller males in both spp., with no difference observed with age of infection. However, in both spp., both female and male worm sizes showed a small but significant (*P* < 0·05) negative linear association with worm load. The lengths of female and male *A. galli* worms decreased by 0·052 cm and 0·0012 cm, respectively, for every single worm increase in the total *A. galli* worm load. Adult *H. gallinarum* lengths followed a similar trend with the rate being 0·014 mm and 0·005 mm, respectively, for every single worm increase in the total worm load.

**Table 4.** Female and male lengths of *A. galli* (cm) and *H. gallinarum* (mm) worms recovered from infected hens killed at different times post-infection

	*Ascaridia galli* (cm/worm)		*Heterakis gallinarum* (mm/worm)	
Weeks post infection	Female	Male	Female	Male
8	8·40 ± 0·56^a^	5·20 ± 0·55^a^	10·8 ± 0·56^a^	8·76 ± 0·50^a^
14	7·56 ± 0·56^a^	4·98 ± 0·55^a^	11·1 ± 0·56^a^	8·62 ± 0·50^a^
20	8·08 ± 0·56^a^	5·14 ± 0·55^a^	10·4 ± 0·56^a^	9·10 ± 0·50^a^
26	7·90 ± 0·56^a^	5·12 ± 0·55^a^	10·5 ± 0·56^a^	8·86 ± 0·50^a^

Values are mean ± SE. Means in the same column sharing common superscripts (a) are not statistically different. SE = standard error.

## Discussion

This study provides the first comparative assessment of *A. galli* and *H. gallinarum* egg distribution across different excreta types in chickens with mono-species ascarid infections, revealing several interesting insights. We found that egg detection is strongly influenced by excreta type, with *A. galli* eggs predominantly present in IE and *H. gallinarum* eggs largely confined to CE. This differentiation was more pronounced for *H. gallinarum* than for *A. galli*, highlighting the much stronger confinement of *H. gallinarum* eggs to CE. These findings have important practical implications, since reliance on mixed or regular intestinal droppings may lead to significant underestimation of *H. gallinarum* infection in the field. Excreta moisture content was similar in both infections and had little impact on EECs. *H. gallinarum* established infections much more efficiently than *A. galli* despite similar infective doses, while worm populations of both species remained stable over time despite continuous larval challenge.

Our first proposition – that in chickens infected with *A. galli* alone, worm eggs would be detected predominantly in the IE rather than in the distinct CE – was supported by the findings. Across all sampling times within the patent period, EECs were consistently and markedly skewed towards IE, being 8-fold higher overall than in CE. This pattern aligns with the biology of *A. galli*, whose primary predilection site in chickens is the small intestine, specifically the anterior half of the jejuno-ileum, where the worms reproduce and lay eggs (Ferdushy et al., 2012). *A. galli* eggs are therefore expected to be shed into the external environment predominantly via the IE. Similarly, in hens mono-infected with *H. gallinarum*, EECs were highly skewed towards CE, strongly supporting our second proposition that eggs from this species would be largely confined to the CE. Our data showed that *Heterakis* eggs were present at a 36-fold higher level in CE than in IE, with only a small fraction detected in the IE, despite IE being produced in much greater volume than CE. This observation is consistent with Fine (1975), who reported that virtually all eggs produced by *H. gallinarum* in the caeca are passed in the caecal fraction of chicken droppings. Unlike Fine (1975), who focused only on *H. gallinarum* eggs in CE, our study provides the first direct quantitative comparison of both *A. galli* and *H. gallinarum* across both excreta types, demonstrating that while *H. gallinarum* eggs are highly confined to CE, *A. galli* eggs are found in both excreta types, but with a significant predominance in the intestinal fraction. Overall, egg distribution is strongly skewed towards the excreta type corresponding to each species’ predilection site, though this skew is far more pronounced for *H. gallinarum*.

In terms of both the proportion of positive samples and EEC levels, *A. galli* eggs were more frequently detected in CE than *H. gallinarum* eggs were in IE. Out of 38 samples of each type examined during patent infections, *A. galli* was detected in more than half of the CE, whereas *H. gallinarum* was detected in less than a quarter of the IE samples tested. Similarly, the mean EPG level of *A. galli* eggs in the CE was about 4-fold higher than the corresponding level of *H. gallinarum* eggs in the IE, with considerably higher range (0–440) in the former. Even though both the IE and CE samples used for egg enumeration were grossly pure, cross-contamination with eggs of either spp. cannot be excluded. Physiologically, CE originates in the paired caecal pouches, where digesta residues undergo microbial fermentation, before being expelled periodically through the cloaca. Intestinal droppings, in contrast, derive from the continuous passage of digesta along the small intestine and colon, mixed with urinary output prior to excretion (King and McLelland, 1984; Scanes, 2021). Importantly, retrograde peristalsis is a normal physiological process in chickens, whereby small intestinal content may intermittently enter the caeca. This provides a plausible mechanism by which *A. galli* eggs, produced in the jejuno-ileum, can reach the caecal lumen and subsequently appear in CE prior to its excretion. Both contents may also mix with other gut contents below the caeca, but *A. galli* eggs are more likely to enter the caeca directly owing to their upstream site of production. The likelihood of detecting *A. galli* eggs in CE is further amplified by their high fecundity (up to 40 000 eggs per female per day; Wongrak et al., 2015), in contrast to the much lower fecundity of *H. gallinarum* (approximately 436 eggs/day; Daş et al., 2014). Together, these physiological processes may explain why *A. galli* eggs are detected at comparatively higher levels and frequencies in CE outside their preferred IE, whereas *H. gallinarum* eggs remain highly confined to CE.

Despite the worm species differences in the degree of separation of eggs into the two excreta types, in practical terms, the EECs in IE appear to best reflect the presence of *A. galli* and those in CE the presence of *H. gallinarum*. Most field samples are IE due to their greater abundance, firmer consistency and ease of collection, which can lead to underestimation of *H. gallinarum* infections. Wongrak et al. (2014), in a longitudinal observation over 2 production years in a free-range farm, reported that 100% of hens harboured *H. gallinarum*, yet eggs were not detected in conventional excreta samples, questioning the reliability of classical egg counts that do not differentiate excreta type or ascarid species. Based on the findings of the current study, CE provides the most accurate indicator for monitoring *H. gallinarum*, and for species-specific EECs, both IE and CE should be collected. Although more labour-intensive, this approach is feasible under field conditions and has been successfully implemented in previous studies (e.g. Shifaw et al., 2023). In the present study, the collection of IE and CE samples was straightforward, with droppings collected throughout the day from floor-based housing, and care taken to avoid collecting from the same bird twice when possible. If excreta samples are uncertain or mixed, they should not be used for species-specific EECs, as this could lead to underestimation of *H. gallinarum* egg output. This limitation was clearly demonstrated in the current study, where ME underestimated EECs compared with pure IE or CE, strongly supporting our third proposition that mixed excreta underestimates EPG in ascarid mono-infections. Overall, EECs from ME were 45% and 60% lower for *A. galli* and *H. gallinarum*, respectively, relative to counts from pure excreta. This reflects the dilution effect caused by the dominance of IE in excreta volume (Van der Klis et al., 1993; Jørgensen et al., 1996; Daş et al., 2011a) combined with the confinement of *H. gallinarum* eggs to CE (Fine, 975).

A practical implication of this study is that in field conditions where co-occurrence of *A. galli* and *H. gallinarum* is very common, examination of random excreta samples (predominantly IE) likely results in false-negative EECs for *H. gallinarum*, even in heavily infected animals (Wongrak et al., 2014). Both for diagnostic and research purposes, the more frequent and common IE or ME are commonly used. These sample types will favour the detection of *A. galli* eggs and underestimate levels of *H. gallinarum* eggs, which are excreted almost exclusively through the CE. We argue that if EECs are to be used to provide more accurate information about the burdens of *A. galli* and *H. gallinarum* in mixed infections egg counts should be performed separately on both IE and CE samples from the flock. In effect, the ratio of EEC in the two excreta types will indicate the relative size of the burden of the two species. The practical usefulness of ME appears to be limited unless used simply as a crude estimate of worm egg load with no speciation. The weak negative association between excreta moisture and EECs observed in this study suggests that watery droppings may slightly dilute egg counts (Le Jambre et al., 2007; Daş et al., 2011b), though the effect was minor and unlikely to impact routine interpretation. Therefore, routine adjustment for moisture content in EEC calculations appears unnecessary.

Another interesting biological finding was the difference in infection establishment rates between the two species. Despite equal infective doses, *H. gallinarum* established infection at more than 10-fold the rate of *A. galli*, indicating far more efficient establishment. Interestingly, the observed EECs of the two species in their preferred excreta type were relatively similar. This can be partly explained by differences in worm size and their contrasting per capita fecundities (Daş et al., 2014; Daş and Gauly, 2014; Wongrak et al., 2015; Feyera et al., 2020). *A. galli* is a larger nematode (Permin and Hansen, 1998), which may increase metabolic demands and limit the number of adults that can be sustained in the host, whereas *H. gallinarum* is smaller, allowing a higher proportion of ingested infective stages to survive and mature, contributing to stable egg outputs. Thus, *H. gallinarum* compensates for its low fecundity with a higher infection establishment rate and smaller body size, whereas *A. galli* relies on high per capita egg production to maintain its population. Together, these traits help explain why EECs of both species appear comparable in the host, despite their contrasting reproductive strategies, and why *H. gallinarum* can persist in poultry flocks despite its lower fecundity.

In both infections, the nematode eggs detected and quantified in different excreta types were outputs from stable adult worm populations in individual birds. Experimental infection, accompanied by continuous reinfection, produced adult worm burdens that remained consistent over time, and the total worms recovered were comparable to levels observed in naturally occurring infections (Thapa et al., 2015; Wuthijaree et al., 2017; Shifaw et al., 2021b, 2023; Feyera et al., 2022). Moreover, worm size similarities imply that all the female worms of the respective spp. were shedding similar numbers of eggs into the excreta (Daş et al., 2011b). The stability of adult worm numbers may be attributed to population equilibrium, where recruitment of new worms balances expulsion or death, with immune-mediated expulsion primarily targeting juvenile stages (Keymer and Slater, 1987; Stehr et al., 2018; Cupo and Beckstead, 2019). Interestingly, despite the higher worm load of *H. gallinarum*, birds infected with *A. galli* showed slightly lower body weight gain, reflecting its greater pathogenic impact on the intestine (Gauly et al., 2001; Kilpinen et al., 2005; Stehr et al., 2019). In contrast, *H. gallinarum*, whose main importance is serving as a vector for *Histomonas* spp., generally causes only mild pathology with minimal effect on performance (Kaushik and Deorani, 1969; Cupo and Beckstead, 2019; McDougald, 2020).

## Conclusions

This study demonstrates that excreta type is a key determinant of ascarid nematode egg detection in chickens, with *A. galli* eggs predominantly found in IE and *H. gallinarum* eggs largely confined to CE. The observed egg distributions and loads reflect the biology of each species and the outputs of stable adult worm populations in an ongoing infection. Effective monitoring, therefore, requires separate EECs for IE and CE: IE counts provide the best measure of *A. galli* burden, while CE counts most reliably indicate *H. gallinarum* infection. Reliance on the more common IE or mixed samples underestimates species-specific burdens, especially for *H. gallinarum*, and risks misleading conclusions. Overall, these findings highlight the importance of species- and excreta-specific EEC monitoring for reliable field diagnosis and control of poultry nematode infections in commercial flocks.
